# Revolutionizing total hip arthroplasty: The role of artificial intelligence and machine learning

**DOI:** 10.1002/jeo2.70195

**Published:** 2025-03-22

**Authors:** Umile Giuseppe Longo, Sergio De Salvatore, Alice Piccolomini, Nathan Samuel Ullman, Giuseppe Salvatore, Margaux D'Hooghe, Maristella Saccomanno, Kristian Samuelsson, Rocco Papalia, Ayoosh Pareek

**Affiliations:** ^1^ Fondazione Policlinico Universitario Campus Bio‐Medico Roma Italy; ^2^ Research Unit of Orthopaedic and Trauma Surgery, Department of Medicine and Surgery Università Campus Bio‐Medico di Roma Roma Italy; ^3^ Orthopedic Unit, Department of Surgery Bambino Gesù Children's Hospital Palidoro, Rome Italy; ^4^ Department of Medicine University of Navarra Pamplona Spain; ^5^ Department of Medical and Surgical Specialties, Radiological Sciences, and Public Health University of Brescia Brescia Italy; ^6^ Department of Bone and Joint Surgery Speciali Civili Brescia Italy; ^7^ Sahlgrenska Sports Medicine Center Gothenburg Sweden; ^8^ Department of Orthopaedics, Institute of Clinical Sciences, Sahlgrenska Academy University of Gothenburg Gothenburg Sweden; ^9^ Department of Orthopaedics Sahlgrenska University Hospital Mölndal Sweden; ^10^ Hospital for Special Surgery New York New York USA

**Keywords:** artificial intelligence, deep learning, machine learning, orthopaedic surgery, total hip arthroplasty

## Abstract

**Purpose:**

There has been substantial growth in the literature describing the effectiveness of artificial intelligence (AI) and machine learning (ML) applications in total hip arthroplasty (THA); these models have shown the potential to predict post‐operative outcomes using algorithmic analysis of acquired data and can ultimately optimize clinical decision‐making while reducing time, cost and complexity. The aim of this review is to analyze the most updated articles on AI/ML applications in THA as well as present the potential of these tools in optimizing patient care and THA outcomes.

**Methods:**

A comprehensive search was completed through August 2024, according to the PRISMA guidelines. Publications were searched using the Scopus, Medline, EMBASE, CENTRAL and CINAHL databases. Pertinent findings and patterns in AI/ML methods utilization, as well as their applications, were quantitatively summarized and described using frequencies, averages and proportions. This study used a modified eight‐item Methodological Index for Non‐Randomized Studies (MINORS) checklist for quality assessment.

**Results:**

Nineteen articles were eligible for this study. The selected studies were published between 2016 and 2024. Out of the various ML algorithms, four models have proven to be particularly significant and were used in almost 20% of the studies, including elastic net penalized logistic regression, artificial neural network, convolutional neural network (CNN) and multiple linear regression. The highest area under the curve (=1) was reported in the preoperative planning outcome variable and utilized CNN. All 20 studies demonstrated a high level of quality and low risk of bias, with a modified MINORS score of at least 7/8 (88%).

**Conclusions:**

Developments in AI/ML prediction models in THA are rapidly increasing. There is clear potential for these tools to assist in all stages of surgical care as well as in challenges at the broader hospital administrative level and patient‐specific level.

**Level of Evidence:**

Level III.

**Abbreviations**
AI: artificial intelligence
ANN: artificial neural network
ASA: American Society of Anesthesiologists
AUC: area under the curve
BMI: body mass index
CNN: convolutional neural network
COPD: chronic obstructive pulmonary disease
CSO: clinically significant outcome
ENPLR: elastic net penalized logistic regression
HJC: hip joint centre
HOA: hip osteoarthritis
HOOS: Hip disability and osteoarthritis outcome score
HRQoL: Health‐related quality of life
JSN: Joint space narrowing
KL: Kellgren and Lawrence
LOS: length of stay
MARS: multivariate adaptive regression splines
MeSH: medical subject headings
METs: metabolic equivalent of task
MINORS: Methodological Index for Non‐Randomized Studies
ML: machine Learning
MLR: multiple linear regression
OECD: Organization for Economic Co‐operation and Development
OST: osteophyte score
PRHS: patient‐reported health state
PRISMA: Preferred Reporting Items for Systematic Reviews and Meta‐analyses
PROM: patient‐reported outcome measure
rHOA: radiographic hip osteoarthritis
SGB: stochastic gradient boosting
SVM: support vector machine
THA: total hip arthroplasty
XGBoost: extreme gradient boosting

## INTRODUCTION

Life expectancy has increased over the past few decades, and total hip arthroplasty (THA) utilization rates have grown accordingly. A 16% increase in THA has been observed in patients aged 65 years or older; this same population has also grown by 12%. Between 2015 and 2050, some European countries, as well as Australia, are projected to experience a 95%–120% increase in THA. The future demand for THA will increase globally, creating a need for modern solutions to healthcare obstacles [31, 32, 33, 35, 36, 45].

Artificial intelligence (AI) broadly refers to the ability of computers and machines to simulate human intelligence, mainly decision‐making and problem‐solving abilities, by using algorithms to process large amounts of data [3]. Machine learning (ML), a branch of AI, is a more autonomous process split into two phases: a teaching phase, where the machine is provided with data to find patterns, and an inference phase, where the machine can infer outcomes based on the previous patterns found [3].

Applying AI's predictive power to THA can ultimately optimize clinical decision‐making while reducing time, cost and complexity [49]. The integration of AI and ML technology into THA can sharpen the field of orthopaedics by enabling more accurate clinical prediction and decision‐making during pre‐ and post‐operative management [33, 44, 46].

AI/ML tools can be applied at an early stage for patient selection and planning, as well as predicting readmission and reoperation risk. During revision arthroplasty preoperative planning, neural networks, a subset of ML, can be used to identify failed implants [6]. At the post‐operative stage, these tools can predict post‐operative complications, patient‐reported outcomes, pain and even prolonged opioid use [12, 34].

As a result, there has been substantial growth in the literature describing the effectiveness of AI and ML applications in THA. These models have shown the potential to predict post‐operative outcomes using algorithmic analysis of acquired data [46]. Lalehzarian et al. reported that decision‐making models could optimize patient satisfaction based on many factors, including age, gender and presence of comorbidities, with the advantage of assessing raw inputs and identifying the most relevant features for analysis [25]. Overall, the utility of AI and ML in orthopaedic surgery continues to expand thanks to the aforementioned benefits as well as the ability to identify patients who are more predisposed to post‐operative complications, thus allowing more targeted therapeutics for better quality outcomes in patients. Given these promising results, it is critical to build an accurate understanding of the implementation of the current models and further develop their applicability in the future [24].

Considering the developments of AI/ML and the increasing demand for THA, it is important to have an up‐to‐date framework of the literature on this topic. Therefore, the purpose of this review is to analyze the most updated articles on AI/ML applications in THA and present the potential of these tools in optimizing patient care and THA outcomes.

## METHODS

### Study selection

The formulation of the research question was done using a PIOS‐approach: Population (P); Intervention (I); Outcome (O) and Study design (S). This systematic review collected and analyzed data on patients who underwent THA (P). The AI/ML methods (I) that processed the patient data to form models which predict preoperative, perioperative and post‐operative outcomes, complications and related costs (O), were reviewed. The following study designs were included (S): retrospective cohort study (RC), prospective cohort study (PC), diagnostic study (DS) and prognostic study (PS).

### Eligibility criteria

Inclusion criteria comprised original clinical studies, including studies which evaluate AI/ML‐based application in clinical decision‐making in hip arthroplasty. Exclusion criteria comprised studies that did not evaluate AI/ML applications in hip arthroplasty, medical imaging analysis studies without explicit reference or application to hip arthroplasty, studies with nonhuman subjects, non‐English‐language studies, inaccessible articles, conference abstracts, reviews and editorials. No limits were placed on the level of evidence or timing of the study because the majority of the reviewed studies were published within the last 10 years.

### Search

A comprehensive search was performed from the inception of the databases included to August 2024, according to the Preferred Reporting Items for Systematic Reviews and Meta‐analyses (PRISMA) guidelines. Publications were searched using the Scopus, Medline, EMBASE, CENTRAL and CINAHL databases, using the following Medical subject headings (MeSH) terms or keywords: ‘hip osteoarthritis’, ‘replacement’, ‘prosthesis’, ‘artificial intelligence’, ‘algorithm’, ‘machine learning’, ‘complications’, ‘blood loss’, ‘blood transfusion’, ‘length of stay’, ‘hospitalisation’, ‘costs’, ‘economic analyses’, ‘functional outcomes’, ‘ROM’, ‘revision’, ‘revision rate’ and ‘surgical technique’. Keywords were used both isolated and combined. Additional studies listed in selected systematic review reference lists were searched. In order to optimize the search, boolean operators (OR and AND) were used.

### Data collection process

**Figure 1:**
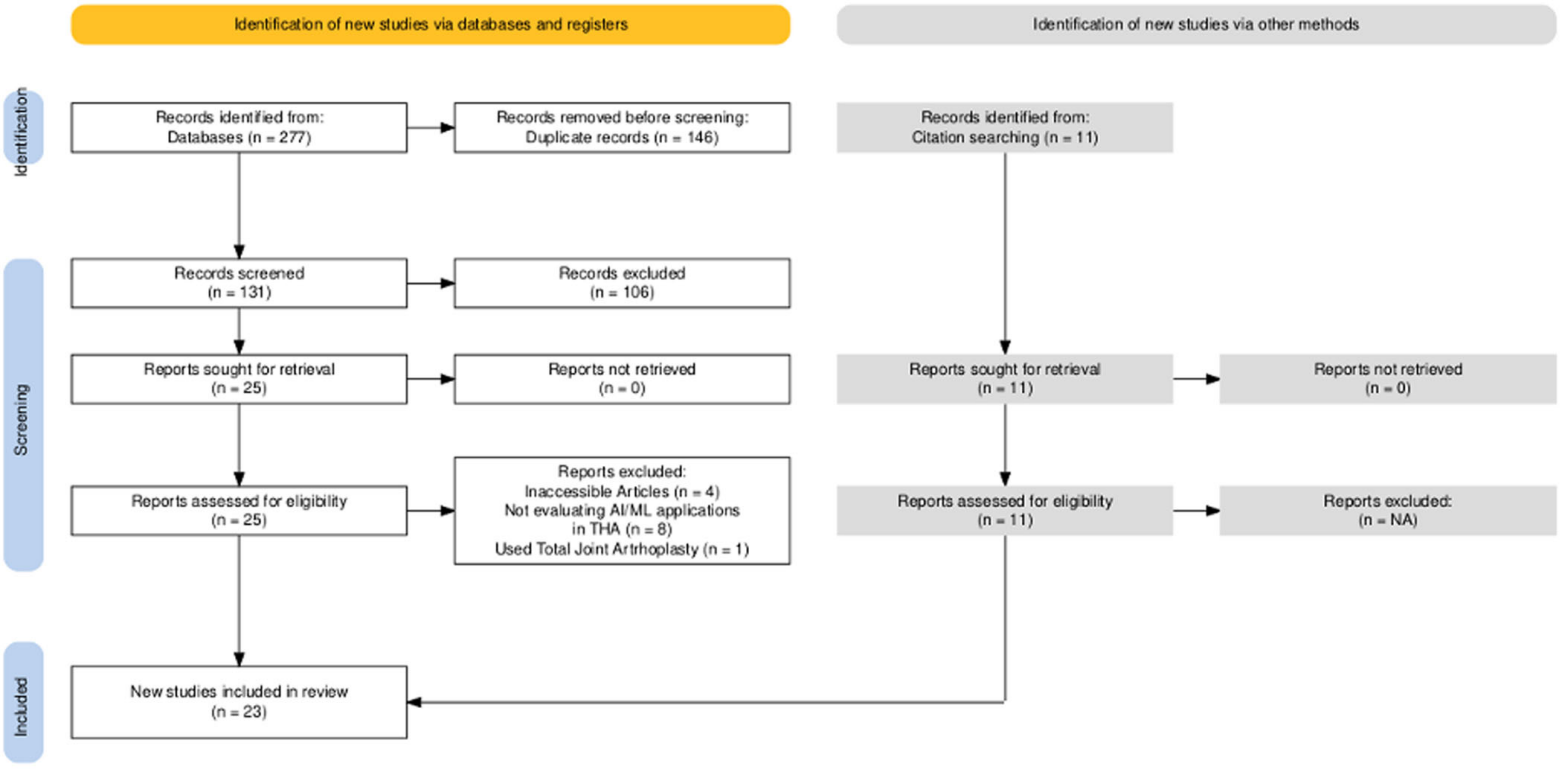
PRISMA diagram for systematic review data collection process. PRISMA, Preferred Reporting Items for Systematic Reviews and Meta‐analyses.

Two independent reviewers (AP and NU) screened each individual article in two stages. Initially, article titles and abstracts were screened, followed by a full‐text review of the selected articles. Any disagreements were resolved by consulting with a third reviewer (SDS). Inclusion and exclusion of the reviewed articles are reported below by the Preferred Reporting Items for Systematic Reviews and Meta‐analyses (PRISMA) flowchart found in Figure 1.

### Data items

General study characteristics of the selected publications were extracted and organized into a database, which included the primary author, year of publication, study design, level of evidence, study duration, AI/ML methods, data sources, input and output variables, sample size of patients in testing sets, average patient age and percentage female patients.

### Data analysis

Pertinent findings and patterns in AI/ML methods utilization, as well as their applications, were quantitatively summarized and described using frequencies, averages and proportions. Brier score and *R* ^2^ values were not included in this review. The reported metrics for each individual AI/ML method, and its corresponding application, were assessed. Metrics used to summarize the performance of reported AI/ML methods include area under the curve (AUC) of receiver operating characteristic curves, accuracy (%), sensitivity (%) and specificity (%).

Input and output variables used in the ML models from the reviewed articles were analyzed as well. Input variables, otherwise known as predictors, are measurable factors which are inputted into the algorithms. These variables can include patient demographics, preoperative clinical data, radiographic measurements and laboratory results. Output variables, also known as target variables or outcomes, are the predictions or results generated by the algorithm. These variables can include post‐operative outcomes, cost prediction and readmission. This system of inputs and outputs allows complex patterns and interdependencies to be found; leveraging these associations then enables the models to predict outcomes of interest when presented with new input data [3].

### Risk of bias assessment

This study used a modified eight‐item Methodological Index for Non‐Randomized Studies (MINORS) checklist for quality assessment. The eight items included: disclosure, study aim, input feature, output feature, validation method, data set distribution, performance metric and AI model. A binary scale was used to score each item: 0 (*not reported or unclear*) and 1 (*reported and clear*). Reviewers AP and NU individually assessed and scored each item. These classifications create a more accurate scoring tool for quality assessments, specifically for publications on diagnostic studies and AI healthcare applications, compared to the original MINORS checklist [26, 51].

## RESULTS

### Search results

A flow‐chart diagram reported the selection process of the studies according to the PRISMA protocol (Figure 1). A total of 277 studies were identified through predefined search terms, with a total of 131 records maintained after duplicate removal. Of those, 106 were excluded from the study through title and abstract screening because they were not inherent to AI/ML applications or not focused on hip joint arthroplasty. Then, 25 full‐text articles were screened. Of these studies, 12 met full inclusion criteria. Through further screening of the bibliography of the included records, 11 additional studies were identified and reported. Ultimately, a total of 23 articles were eligible for this study.

### Study characteristics

The majority of the articles were retrospective cohort studies, accounting for over 68% of the cohort, however prognostic studies (5%), prospective cohort studies (21%) and diagnostic studies (5%) were also included. Level of evidence of the reviewed studies ranged from I to IV. Of the selected studies, the following levels of evidence and study designs were included: 1 Level I prognostic study, 3 Level II prospective studies, 17 Level III retrospective studies and 2 Level IV diagnostic study. The selected studies were published between 2016 and 2024. The average number of patients included in the testing model was 10,738 (standard deviation [SD] = 20.45), with an average patient age of 62.6 years. On average, 61.2% of the patients were females. The study by Ramkumar et al. reported the highest number of patients (*n* = 78,335) [47]. Of the 20 studies that were analyzed in this review, 15 were single‐centred studies, while 5 were multicentered. The aforementioned data can be found in Table 1.

**Table 1:** Study characteristics.

Authors and Year	Country	Study design	Level of evidence	Follow‐up	Sample size	Mean patient age	% Female patients
Alam et al., 2019 [1]	UK	RCS	III	–	10	–	–
Borjali et al., 2020 [5]	USA	RCS	III	1 year	25	61.3	47%
Chen et al., 2023 [7]	Taiwan	DS	IV	3 months	4643	–	–
Crawford et al., 2023 [8]	USA	RCS	III	4 months	158	65	60.8%
Gabriel et al., 2019 [10]	USA	RCS	III	2 years	240	–	50.50%
Gielis et al., 2019 [11]	Netherlands	RCS	III	3 years	1044	55.9 (±5.1)	87.30%
Hirvasniemi et al., 2019 [14]	Netherlands	PCS	II	10 years	197	55.7 (±5.2)	83.80%
Hyer et al., 2020 [15]	USA	RCS	III	2 years	19,522	–	–
Jang et al., 2022 [16]	USA	RCS	III	10 years	4796	31	58%
Jang et al., 2022 [17]	USA	DS	IV	11 years	793	61.4	57.60%
Jodeiri et al., 2020 [18]	Japan	PCS	II	–	95	–	–
Jones et al., 2019 [19]	USA	RCS	III	1 year	43,407	–	–
Kang et al., 2020 [20]	South Korea	RCS	III	–	1202	–	–
Karhade et al., 2019 [21]	USA	RCS	III	18 years	1101	66 (IQR 57–74)	49.50%
Klemt et al., 2022 [22]	USA	RCS	III	3 years	183	62 (IQR 54–71)	57.30%
Kunze et al., 2020 [23]	USA	RCS	III	3 years	183	62 (IQR 54–71)	57.30%
Nemes et al., 2016 [43]	Sweden	RCS	III	1 years	646	68.9	58.40%
Ramkumar et al., 2019 [47]	USA	RCS	III	4 years	78,335	75.3 (65–106)	63.60%
Ramkumar et al., 2019 [48]	USA	RCS	III	4 years	30,584	–	–
Sherafati et al., 2020 [49]	USA	RCS	II	4 years	78	63.1 (±8.5)	47%
Sniderman et al., 2020 [51]	Canada	PS	I	3 months	53	66.7 (±9.7) (34–84)	44%
Van de Meulebroucke et al., 2019 [52]	Belgium	RCS	III	1 year	100	–	–
Zhang et al., 2021 [56]	Singapore	PCS	II	12 years	302	62.9 (±12.1)	69.90%

Abbreviations: DS, diagnostic study; PCS, prospective cohort study; PS, prognostic study; RCS, retrospective cohort study.

Out of the various ML algorithms, four models have proven to be particularly significant and were used in almost 20% of the studies: elastic net penalized logistic regression (ENPLR), artificial neural network (ANN), convolutional neural network (CNN) and multiple linear regression (MLR).

## OUTCOME VARIABLES

The following sections are the outcome variables that are reported in Table 2.

**Table 2:** Input and output variables (OV).

Authors and Year	Input variables			OV		OV2	
Alam et al., 2019 [1]	Primary prostheses cost, revision procedures cost, HRQoL, state‐to‐state transition probabilities			Cost prediction		–	
Borjali et al., 2020 [5]	X‐ray			Preoperative planning		–	
Chen et al., 2023 [7]	X‐ray			Total Hip Arthroplasty prediction		–	
Crawford et al., 2023 [8]	Age, sex, BMI, race, CCI, diabetes, COPD, CKD, depression, opioid use, benzodiazepine use, smoking status, degree of arthritis, physical therapy			Total Hip Arthroplasty prediction		–	
Gabriel et al., 2019 [10]	Age, preoperative opioid use, mets, sex, preoperative anaemia, COPD, hypertension, obesity, primary anaesthesia type			LOS		–	
Gielis et al., 2019 [11]	Demographics, clinical examination, radiographic parameters, incident HOA risk			Clinical outcomes (rhoa)		–	
Hirvasniemi et al., 2019 [14]	Age, gender, BMI, baseline KL grade, bone texture			Preoperative planning		Clinical Outcomes (rhoa, JSN, OST)	
Hyer et al., 2020 [15]	Age, sex, comorbidity, cost, LOS			Cost prediction		–	
Jang et al., 2023 [16]	Demographics, clinical examination, radiographic parameters, socioeconomic factors			Predictive model for 10‐year THA risk		Clinical outcomes	
Jang et al., 2022 [17]	X‐ray			Preoperative planning		Clinical outcomes (HJC)	
Jodeiri et al., 2020 [18]	X‐ray			Preoperative planning		–	
Jones et al., 2019 [19]	Comorbidity, LOS			Readmission		Revision	
Kang et al., 2020 [20]	X‐ray			Preoperative planning		–	
Karhade et al, 2019 [21]	Age, opioid exposure duration, preoperative haemoglobin, preoperative medications			Preoperative planning		Functional Outcomes (Prolonged post‐operative opioid prescription)	
Klemt et al., 2022 [22]	ASA score, BMI, revision surgery indication, bearing surface, female gender, implant fixation			Revision		–	
Kunze et al., 2020 [23]	Demographics, medical history, PRHS, modified Harris Hip Score			Functional outcomes (CSO)		–	
Nemes et al., 2016 [43]	Preoperative EQ‐5D score, EQ visual analogue scale, visual analogue scale pain, Charnley classification, age, gender, BMI, surgical approach prosthesis type			Clinical outcomes (HRQoL)		–	
Ramkumar et al., 2019 [47]	Age, sex, comorbidity, readmission			LOS		Cost prediction	
Ramkumar et al., 2019 [48]	Age, sex, comorbidity, readmission			LOS		Cost prediction	
Sherafati et al., 2020 [49]	Biomarkers (Necrosis score, Co and Cr metal‐ion serum levels, synovial volume and thickness, mscore (metallosis), off‐resonance frequency density)			Preoperative planning		–	
Sniderman et al., 2020 [51]	Patient demographics, PRHS, cognitive appraisal processes, surgical approach			Functional outcome (HOOS)		–	
Van de Meulebroucke et al., 2019 [52]	THA implant characteristics, Intraoperative complications, post‐operative complications, interventions			Cost prediction		Preoperative planning	
Zhang et al., 2021 [56]	Patient demographics, comorbidities, preoperative proms			Clinical outcome (patient satisfaction)		–	

Authors and Year	Country	Study design	Level of evidence	Follow‐up	Sample size	Mean patient age	% Female patients
Alam et al., 2019 [1]	UK	RCS	III	–	10	–	–
Borjali et al., 2020 [5]	USA	RCS	III	1 year	25	61.3	47%
Chen et al., 2023 [7]	Taiwan	DS	IV	3 months	4643	–	–
Crawford et al., 2023 [8]	USA	RCS	III	4 months	158	65	60.8%
Gabriel et al., 2019 [10]	USA	RCS	III	2 years	240	–	50.50%
Gielis et al., 2019 [11]	Netherlands	RCS	III	3 years	1044	55.9 (±5.1)	87.30%
Hirvasniemi et al., 2019 [14]	Netherlands	PCS	II	10 years	197	55.7 (±5.2)	83.80%
Hyer et al., 2020 [15]	USA	RCS	III	2 years	19,522	–	–
Jang et al., 2023 [16]	USA	RCS	III	10 years	4796	31	58%
Jang et al., 2022 [17]	USA	DS	IV	11 years	793	61.4	57.60%
Jodeiri et al., 2020 [18]	Japan	PCS	II	–	95	–	–
Jones et al., 2019 [19]	USA	RCS	III	1 year	43,407	–	–
Kang et al., 2020 [20]	South Korea	RCS	III	–	1202	–	–
Karhade et al, 2019 [21]	USA	RCS	III	18 years	1101	66 (IQR 57–74)	49.50%
Klemt et al., 2022 [22]	USA	RCS	III	3 years	183	62 (IQR 54–71)	57.30%
Kunze et al., 2020 [23]	USA	RCS	III	3 years	183	62 (IQR 54–71)	57.30%
Nemes et al., 2016 [43]	Sweden	RCS	III	1 year	646	68.9	58.40%
Ramkumar et al., 2019 [47]	USA	RCS	III	4 years	78,335	75.3 (65–106)	63.60%
Ramkumar et al., 2019 [48]	USA	RCS	III	4 years	30,584	–	–
Sherafati et al., 2020 [49]	USA	RCS	II	4 years	78	63.1 (±8.5)	47%
Sniderman et al., 2020 [51]	Canada	PS	I	3 months	53	66.7 (±9.7) (34–84)	44%
Van de Meulebroucke et al., 2019 [52]	Belgium	RCS	III	1 years	100	–	–
Zhang et al., 2021 [56]	Singapore	PCS	II	12 years	302	62.9 (±12.1)	69.90%

Abbreviations: ASA, American Society of Anesthesiologists; BMI, body mass index; COPD, chronic obstructive pulmonary disease; CSO, clinically significant outcome; HJC, hip joint centre; HOA, hip osteoarthritis; HOOS, Hip disability and Osteoarthritis Outcome Score; HRQoL, health‐related quality of life; IQR, interquartile range; JSN, joint space narrowing; KL, Kellgren and Lawrence; LOS, length of stay; MET, metabolic equivalent task; OST, osteophyte score; PRHS, patient‐reported outcome measure; THA, total hip arthroplasty.

Under each outcome variable, there is included the specific ML methods used to predict the outcome variables, prediction model performance metric values and how many studies developed prediction models for the specific outcome variable, all of which are reported in Table 3.

**Table 3:** AI/ML characteristics.

Authors and Year	AI/ML methods		Data sources		AUC		Accuracy	Sensitivity		Specificity
Alam et al., 2019 [1]	ANN		Multicenter		–		–	–		–
Borjali et al., 2020 [5]	CNN		Single‐centre study		–		1	–		–
Chen et al., 2023 [7]	CNN		Single‐centre study		0.994		0.977	0.920		0.992
Crawford et al., 2023 [8]	SGB		Multicenter study		0.83		–	–		–
Gabriel et al., 2019 [10]	MLR		Single‐centre study		0.735		–	64.5		72.1
Gielis et al., 2019 [11]	ENPLR		CHECK cohort		0.86		–	–		–
Hirvasniemi et al., 2019 [14]	ENPLR		CHECK cohort		0.71		–	–		–
Hyer et al., 2020 [15]	decision tree		Medicare		–		83.40%	–		–
Jang et al., 2023 [16]	GA2M		Osteoarthritis Initiative		0.81		–	–		–
Jang et al., 2022 [17]	CNN		Osteoarthritis Initiative (Multicenter)		–		91%	–		–
Jodeiri et al., 2020 [18]	CNN		Single‐centre study		–		–	–		–
Jones et al., 2019 [19]	Logistic regression		Medicare		0.66		–	–		–
Kang et al., 2020 [20]	CNN		Multicenter		0.99		–	–		–
Karhade et al, 2019 [21]	SGB		Multicentre		0.77		–	–		–
	random forest				0.75		–	–		–
	SVM				0.59		–	–		–
	ANN				0.77		–	–		–
	ENPLR				0.77		–	–		–
Klemt et al., 2022 [22]	ANN		Single‐centre study		0.85		–	–		–
	SGB			0.81				
	random forest			0.8				
	ENPLR			0.81				
Kunze et al., 2020 [23]	SGB		Single‐centre study		0.94		–	–		–
	random forest				0.97		–	–		–
	SVM				0.87		–	–		–
	ANN				0.92		–	–		–
	ENPLR				0.87		–	–		–
Nemes et al., 2016 [43]	MARS		Swedish Hip Arthroplasty Register		–		–	–		–
	Linear regression			–		–	–		–
Ramkumar et al., 2019 [47]	ANN		National Inpatient Sample		0.82 (LOS)		0.75 (LOS)	–		–
Ramkumar et al., 2019 [48]	Bayesian		Patient database		0.87 (LOS)		–	–		–
Sherafati et al., 2020 [49]	ANN		Single‐centre study		0.84		–	–		–
Sniderman et al., 2020 [51]	LASSO		Single‐centre study		–		–	–		–
Van de Meulebroucke et al., 2019 [52]	ANN		Single‐centre study		–		0.95	–		–
Zhang et al., 2021 [56]	Random forest		Single‐centre study		0.68		–	53.00%		76.50%
	XGBoost				0.66		–	50.50%		76.50%
	SVM				0.74		–	57.90%		76.50%
	logistic LASSO				0.76		–	65.30%		82.40%

Authors and Year	Input variables				Output variables (OV)			OV2		
Alam et al., 2019 [1]	Primary prostheses cost, revision procedures cost, HRQoL, state‐to‐state transition probabilities				Cost prediction			–		
Borjali et al., 2020 [5]	X‐ray				Preoperative planning			–		
Chen et al., 2023 [7]	X‐ray				Total Hip Arthroplasty prediction			–		
Crawford et al., 2023 [8]	Age, sex, BMI, race, CCI, diabetes, COPD, CKD, depression, opioid use, benzodiazepine use, smoking status, degree of arthritis, physical therapy				Total Hip Arthroplasty prediction			–		
Gabriel et al., 2019 [10]	Age, preoperative opioid use, mets, sex, preoperative anaemia, COPD, hypertension, obesity, primary anaesthesia type				LOS			–		
Gielis et al., 2019 [11]	Demographics, clinical examination, radiographic parameters, incident HOA risk				Clinical outcomes (rhoa)			–		
Hirvasniemi et al., 2019 [14]	Age, gender, BMI, baseline KL grade, bone texture				Preoperative planning			Clinical outcomes (rhoa, JSN, OST)		
Hyer et al., 2020 [15]	Age, sex, comorbidity, cost, LOS				Cost prediction			–		
Jang et al., 2023 [16]	Demographics, clinical examination, radiographic parameters, socioeconomic factors				Predictive model for 10‐year THA risk			Clinical outcomes		
Jang et al., 2022 [17]	X‐ray				Preoperative planning			Clinical outcomes (HJC)		
Jodeiri et al., 2020 [18]	X‐ray				Preoperative planning			–		
Jones et al., 2019 [19]	Comorbidity, LOS				Readmission			Revision		
Kang et al., 2020 [20]	X‐ray				Preoperative planning			–		
Karhade et al, 2019 [21]	Age, opioid exposure duration, preoperative haemoglobin, preoperative medications				Preoperative planning			Functional Outcomes (Prolonged post‐operative opioid prescription)		
Klemt et al., 2022 [22]	ASA score, BMI, revision surgery indication, bearing surface, female gender, implant fixation				Revision			–		
Kunze et al., 2020 [23]	Demographics, medical history, PRHS, modified Harris Hip Score				Functional outcomes (CSO)			–		
Nemes et al., 2016 [43]	Preoperative EQ‐5D score, EQ visual analogue scale, visual analogue scale pain, Charnley classification, age, gender, BMI, surgical approach prosthesis type				Clinical outcomes (HRQoL)			–		
Ramkumar et al., 2019 [47]	Age, sex, comorbidity, readmission				LOS			Cost prediction		
Ramkumar et al., 2019 [48]	Age, sex, comorbidity, readmission				LOS			Cost prediction		
Sherafati et al., 2020 [49]	Biomarkers (Necrosis score, Co and Cr metal‐ion serum levels, synovial volume and thickness, mscore (metallosis), off‐resonance frequency density)				Preoperative planning			–		
Sniderman et al., 2020 [51]	Patient demographics, PRHS, cognitive appraisal processes, surgical approach				Functional outcome (HOOS)			–		
Van de Meulebroucke et al., 2019 [52]	THA implant characteristics, Intraoperative complications, post‐operative complications, interventions				Cost prediction			Preoperative planning		
Zhang et al., 2021 [56]	Patient demographics, comorbidities, preoperative proms				Clinical outcome (patient satisfaction)			–		

Authors and Year	Country	Study design		Level of evidence	Follow‐up	Sample size		Mean patient age	% Female patients	
Alam et al., 2019 [1]	UK	RCS		III	–	10		–	–	
Borjali et al., 2020 [5]	USA	RCS		III	1 year	25		61.3	47%	
Chen et al., 2023 [7]	Taiwan	DS		IV	3 months	4643		–	–	
Crawford et al., 2023 [8]	USA	RCS		III	4 months	158		65	60.8%	
Gabriel et al., 2019 [10]	USA	RCS		III	2 years	240		–	50.50%	
Gielis et al., 2019 [11]	Netherlands	RCS		III	3 years	1044		55.9 (±5.1)	87.30%	
Hirvasniemi et al., 2019 [14]	Netherlands	PCS		II	10 years	197		55.7 (±5.2)	83.80%	
Hyer et al., 2020 [15]	USA	RCS		III	2 years	19,522		–	–	
Jang et al., 2023 [16]	USA	RCS		III	10 years	4796		31	58%	
Jang et al., 2022 [17]	USA	DS		IV	11 years	793		61.4	57.60%	
Jodeiri et al., 2020 [18]	Japan	PCS		II	–	95		–	–	
Jones et al., 2019 [19]	USA	RCS		III	1 year	43,407		–	–	
Kang et al., 2020 [20]	South Korea	RCS		III	–	1202		–	–	
Karhade et al, 2019 [21]	USA	RCS		III	18 years	1101		66 (IQR 57‐74)	49.50%	
Klemt et al., 2022 [22]	USA	RCS		III	3 years	183		62 (IQR 54–71)	57.30%	
Kunze et al., 2020 [23]	USA	RCS		III	3 years	183		62 (IQR 54–71)	57.30%	
Nemes et al., 2016 [43]	Sweden	RCS		III	1 year	6,46		68.9	58.40%	
Ramkumar et al., 2019 [47]	USA	RCS		III	4 years	78,335		75.3 (65–106)	63.60%	
Ramkumar et al., 2019 [48]	USA	RCS		III	4 years	30,584		–	–	
Sherafati et al., 2020 [49]	USA	RCS		II	4 years	78		63.1 (±8.5)	47%	
Sniderman et al., 2020 [51]	Canada	PS		I	3 months	53		66.7 (±9.7) (34–84)	44%	
Van de Meulebroucke et al., 2019 [52]	Belgium	RCS		III	1 years	100		–	–	
Zhang et al., 2021 [56]	Singapore	PCS		II	12 years	302		62.9 (±12.1)	69.90%	

Abbreviations: ANN, artificial neural network; AUC, area under the curve; BMI, body mass index; CNN, convolutional neural network; ENPLR, elastic net penalized logistic regression; GA2M, Generalized Additive Model with Pairwise Interactions; IQR, interquartile range; MARS, multivariate adaptive regression splines; MLR, multiple linear regression; SGB, stochastic gradient boosting; SGM, Stochastic Gradient Boosting; SVM, support vector machine; XGBoost, extreme gradient boosting.

### Risk of THA

Two studies explored the necessity of THA in patients with OA based on preoperative radiographs [7, 8].

### Preoperative planning

Four of the seven studies reporting this outcome relied on CNN, one of which had an accuracy of 1, while the other studies used ENPLR, ANN and stochastic gradient boosting (SGB) reporting an AUC of 0.71, 0.84 and 0.77, respectively [5, 7, 14, 16, 17, 19, 20, 50, 53].

### Cost prediction

Three of the five studies reporting this outcome variable used ANN, one of which reported an AUC of 0.82; however, SBG and Bayesian were also used, which reported an AUC of 0.87 [1, 15, 47, 48, 53].

### Length of stay (LOS)

Three studies analyzed this outcome, using ANN, Bayesian and MLR, reporting an AUC of 0.82 and 0.87, respectively [10, 47, 48].

### Readmission

Only one study analyzed this variable together with the revision outcome, using logistic regression. The reported AUC value was 0.66 [18].

### Revision

Two studies reporting this outcome variable used ANN, SGB, random forest, ENPLR and logistic regression. The more powerful model was ANN, with an AUC of 0.85 [18, 21].

### Other complications

One study reported this outcome variable, using ANN, with an accuracy of 0.95 [53].

### Functional outcomes

Two studies reporting this outcome variable used SGB, random forest, SVM, neural network and ENPLR, with an average AUC of 0.82. SGB and random forest were the more powerful methods, with AUC averages of 0.86. The third study used LASSO, reporting an *R* ^2^ value of 0.29 [20, 22, 52].

### Clinical outcome

Of the five studies reporting clinical outcomes, two used ENPLR with an average AUC of 0.79. One study used MARS and linear regression, with *R* ^2^ values of 0.158 and 0.157, respectively, while the stacked model had a more powerful *R* ^2^ value of 0.17. The fourth study used four methods; of these, LASSO and SVM with respective AUC values of 0.76 and 0.74 had the highest predictive values. The final study used CNN with an accuracy of 91% [11, 14, 16, 42, 57].

## MINORS RESULTS

**Figure 2:**
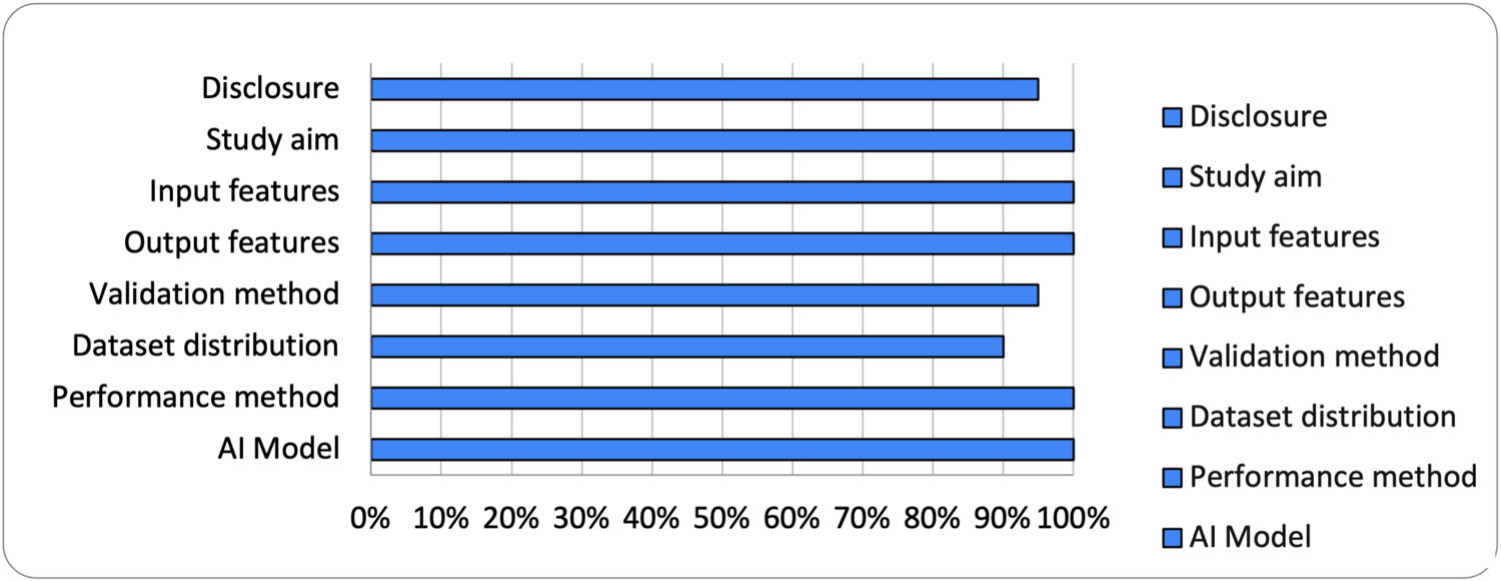
Modified methodological index for nonrandomized studies (MINORS).

A total of 23 studies were included in our review, all were assessed to check for quality and risk of bias according to the modified MINORS method. With the exception of one, which did not clearly describe its validation method or data set distribution resulting in potentially biased outcomes, all 23 studies demonstrated a high level of quality and low risk of bias, with a modified MINORS score of at least 7/8 (88%), as reported in Figure 2.

## DISCUSSION

This systematic review analyzed the current state of AI/ML prediction models in THA, extracting the relevant data to demonstrate the models' potential in improving clinical decision‐making, preoperative planning, related costs, complications, and ultimately patient outcomes. The primary aim of this review is to evaluate the effectiveness of AI/ML applications in THA compared to traditional methods in predicting outcomes, improving clinical decision‐making, and optimizing healthcare delivery. In doing so, we also describe the current landscape of AI/ML applications in the field, identifying both strengths and limitations. A key finding was the outstanding discriminative ability of CNN prediction models in identifying failed implant design in preoperative planning of revision THA. Previous research has shown that 10% of failed implant designs could not be identified preoperatively, as well as 2% intra‐operatively, and projected that by 2030, cumulative surgeon time to identify failed implants would rise to almost 130,000 h (about 15 years) while opportunity cost of surgeon as well as staff time would double compare to 2020 [38, 55, 56]. These changes can lead to increased operating room procedure time, costs, blood loss and recovery time [55, 56]. As the CNN model's singular input variable was anteroposterior radiographs, this model can potentially be a low cost, timesaving, accurate detection tool ubiquitous in revision THA clinical settings.

From a larger perspective, AI/ML models performed outstandingly in predicting CSO post‐THA and performed excellently in predicting LOS, rHOA and periprosthetic tissue necrosis progression. In predicting CSO post THA and LOS, AI/ML models leveraged modifiable risk factors to directly identify patients at risk for suboptimal outcomes as well as indirectly provide healthcare providers with actionable risk factors to optimize outcomes at the preoperative stage and facilitate shared decision‐making. Preoperative opioid use and body mass index are two modifiable risk factors that have been found to predispose patients to lower patient‐reported outcomes post‐THA [4, 22]. By using random forest, a supervised ML algorithm, THA patients at greater risk for poor CSO in post‐operative PROM can be identified, allowing surgeons to intervene preoperatively and potentially minimize risks [22, 29]. Due to the increasing THA utilization, finding ways to prevent prolonged LOS is important to contain hospital costs and mitigate readmission risk [40, 54]. Pain and physical therapy are two modifiable causes of prolonged LOS; by using ANN and Bayesian prediction models to identify patients at higher risk for prolonged LOS, clinicians can potentially more effectively allocate hospital resources, and decrease hospital costs, by targeting these two factors in higher‐risk patients [10, 39].

### Preoperative applications

AI/ML models prove to be a useful predictive tool; two such models are CNN and ENPLR, which have exhibited great potential in optimizing clinical and surgical decision‐making at the preoperative stage. These two models can serve as cost‐effective solutions to escalating healthcare expenses, while simultaneously paving the way for more personalized care and potentially reduced OR (operating room) time [56]. CNN‐based implant recognition programs have proven to be a novel automatic approach to the collection and identification of failed implant designs. Drawing on CNN predictive power can make manual identification of implant devices less time‐consuming and less error‐prone, thus reducing OR time and overall healthcare costs while increasing revision surgery success [5, 19]. CNN models can be implemented to analyze preoperative data to calculate patients' risk for post‐operative complications based on the patients' medical history and needs, further providing a virtual guide to optimize implant placement to achieve better alignment and stability [16, 17]. ENPLR‐based algorithms may be useful in predicting the personalized risk of incident rHOA and THA based on radiographic findings [11, 14, 28]. Overall, the precision and effectiveness of preoperative care in THA can be significantly improved by AI and ML models, leading to better patient outcomes and reduced healthcare expenses. Chen and Crawford explored the possibility of predicting the risk of THA in a cohort of patients using ML. AI could help researchers to early identify patients who can benefit from THA replacement, minimizing the risk of complex surgeries due to excessive latency of the treatment.

### Post‐operative applications

AI/ML models may prove advantageous at the post‐operative stage as well, by utilizing their predictive capabilities clinicians may better prevent or manage post‐operative complications, thus limiting adverse events, as well as forecast surgical outcomes and readmission risk. Misuse and overdosing of prescription opioids are a significant burden on public health systems globally in regard to both mortality and costs; therefore, efficacious preventive measures are needed [2, 27, 43]. ENPLR algorithms have demonstrated the ability to identify patients who are at higher risk for prolonged post‐operative opioid prescriptions. The early identification and intervention in high‐risk cases could help prevent or alleviate the unfavourable outcomes of opioid dependence [20]. In cases of high projected risk of potentially severe post‐operative complications and outcomes, these tools may provide a useful guide to better customize therapy for patients. MARS models may allow for predicted HRQoL, which can be incorporated into the decision‐making between providers and patients, enabling a review of optimal treatment options and expected benefits following THA [42]. Finally, ANN and Bayesian models can be useful in identifying patients who are more likely to need prolonged LOS, essentially providing a functional tool to achieve strategic planning for hospital bed availability [10]. In short, AI/ML tools have the potential to improve post‐operative care in THA by reducing costs while increasing targeted and personalized therapy based on patients' unique characteristics, thus resulting in enhanced patient satisfaction and overall healthcare [18, 30, 42, 48, 50, 52, 53].

ML methods exhibit a strong potential in optimizing patient outcomes by contributing to each stage of THA, but many models still require improvements before they can become standardized and widely adopted in external institutions, as well as different geographic regions [13, 23]. Among the studies reviewed, the most common and salient constraints in model performance and generalizability were limited data set size, limited data granularity and lack of external validation. Approximately 50% of the reviewed articles used single‐centre data sources; as AI models are shaped by the data inputted, the limited representation does not account for regional differences or more varied patient characteristics and thus constrains the model's usability. Access to larger data sets across distinct populations and regions is paramount to preventing overfitting or drawing conclusions that are only applicable to identically distributed data sets [9, 41]. Data granularity relates to the extent of detail in the data; by increasing it, the output variables can become more precise and thus more effective in clinical predictions and decision‐making. Larger studies can potentially curtail the limited data set issue while making available potentially important predictors, such as relevant clinical, socioeconomic, and administrative factors [10, 17, 18, 20, 47]. Finally, collaboration among researchers and their institutions will be required to optimize data sharing and model reliability [13, 16, 20, 22, 47, 48, 50, 57].

## LIMITATIONS

This review does have some potential limitations. Additionally, most developed models did not undergo the relevant testing and validation that is needed to evaluate their clinical efficacy; therefore, conclusions are limited to the potential of these models. It should also be mentioned that one study reported a CNN model with an accuracy of 1, which may indicate, despite the prior highlighted strengths, some weaknesses such as overfitting, although it was reported that steps were taken to reduce this, or a small data set [15]. Lopez et al. reviewed the accuracy of AI/ML applications in THA as well as total knee arthroplasty; the present review is a more precise and exact study focusing solely on THA [37]. The reviewed ML models were formulated for highly specific clinical tasks in the realm of THA; thus, this review presented the exact AI/ML methods used as well as their unique input and output variables, which are the foundations of these models and their clinical applicability. Furthermore, a quality assessment was performed using a modified version of the MINORS checklist. This modified version was developed by Langerhuizen et al. as a response to the lack of a suitable tool for bias assessment in diagnostic studies [26, 51]. Risk of bias assessments must be incorporated in future reviews to help solidify the legitimacy and integrity of the reviewed models. This systematic review did not perform a meta‐analysis and, therefore, did not report statistical significance. This is due to a high level of heterogeneity among the articles included. The studies included a broad pool of different populations, interventions, algorithms and outcomes, rendering a meta‐analysis inapplicable.

Different levels of evidence were included in the paper, providing high heterogeneity between articles in terms of scientific validity. Finally, one major limitation of current AI/ML applications in THA is the lack of external validation across diverse populations and healthcare settings. Furthermore, the reliance on short follow‐up periods (<12 months in most studies) restricts the ability to assess long‐term clinical and economic outcomes. Future research should prioritize standardized validation protocols and multi‐year follow‐ups to enhance the robustness and clinical utility of these models.

## CONCLUSIONS

AI/ML has the potential to aid surgical care at all stages and broadly optimize healthcare, from hospital administration to individual patient care. Models demonstrated impressive predictive abilities, but standardized implementation remains to be seen. In order to move beyond the theoretical potential of these models, clinical efficacy and standardization must be proven and dealt with. It is essential for future developments to perform external validation as well as assessments such as calibration and decision curve analysis to verify clinical utility. Researchers must ensure that standardized, comprehensive, and high‐quality data is used, as ML models are only as good, and unbiased, as the data fed into them. Mainstream prediction models have the luxury of dealing with lesser implications; as the medical field deals with human lives, the accuracy and integrity strived for in surgical interventions should be matched in the orthopaedics ML developments.

## AUTHOR CONTRIBUTIONS

*Manuscript preparation, study design, database interpretation and manuscript revision*: Umile Giuseppe Longo, Sergio De Salvatore and Alice Piccolomini. *Manuscript preparation, database interpretation and statistical analysis*: Nathan Samuel Ullman, Alice Piccolomini and Rocco Papalia. *Manuscript preparation, figures and tables preparation, study design*: Alice Piccolomini, Sergio De Salvatore and Giuseppe Salvatore. *Manuscript preparation and database interpretation*: Umile Giuseppe Longo, Nathan Samuel Ullman, Sergio De Salvatore and Maristella Saccomanno. *Study design, manuscript revision*: Umile Giuseppe Longo, Margaux D'Hooghe and Kristian Samuelsson. The authors read and approved the final manuscript.

## CONFLICT OF INTEREST STATEMENT

Kristian Samuelsson is a member of the Board of Directors of Getinge AB (publ). The other authors declare no conflicts of interest.

## ETHICS STATEMENT

The ethics statement is not available.

## Data Availability

The data sets used and/or analyzed during the current study available from the corresponding author on reasonable request.
